# Designed peptide increases clearance of Aβ toxic oligomers and inhibits plaque formation

**DOI:** 10.1002/alz70859_104011

**Published:** 2025-12-26

**Authors:** Carolyn Tallon, Sebastien Pradel, Koya Yakabi, Emma Breyak, Chandresh Gajera, Jeff Posakony, Gil Block, Valerie Daggett

**Affiliations:** ^1^ AltPep Corporation, Seattle, WA USA

## Abstract

**Background:**

Recent advancements in the understanding of the molecular pathophysiology of amyloid‐β (Aβ) in Alzheimer’s disease (AD) have identified soluble oligomers as the dominant toxic form of Aβ correlating with cognitive decline. These toxic oligomers have been observed to adopt a nonstandard secondary structure: α‐sheet. SOBIN‐01, has been specifically designed to target the α‐sheet structure of toxic oligomers.

**Method:**

Using biolayer interferometry we determined the binding affinity of SOBIN‐01 to the monomeric random coil, α‐sheet, and β‐sheet forms of Aβ. We then examined the efficacy of fluorescently labeled α‐sheet Aβ uptake in vitro in cultured microglia and macrophage cells with SOBIN‐01 treatment. Finally, we measured the in vivo effects of SOBIN‐01 on Aβ plaque accumulation by treating 3‐month‐old Tg2576 AD mice for 12 months.

**Result:**

The biolayer interferometry studies demonstrated that SOBIN‐01 binds tightly and preferentially to α‐sheet oligomers over both random coil monomers and β‐sheet protofibrils, with a 2.4x10^4^‐ and 1.2x10^5^‐fold change, respectively. We observed enhanced phagocytosis of fluorescently labeled α‐sheet Aβ in a dose‐dependent manner from 0.1‐10 μM SOBIN‐01 in both microglia and macrophages, with a 1.9‐ and 3.4‐fold change with 10 μM treatment, respectively. We then examined whether SOBIN‐01 would have effects on Aβ accumulation in the Tg2576 AD mouse model and observed a significant reduction in the percent plaque area in the brains of SOBIN‐01 treated animals.

**Conclusion:**

SOBIN‐01 is a highly specific peptide that targets the α‐sheet conformation of Aβ and leads to enhanced phagocytosis by microglia and macrophage cells. In vivo SOBIN‐01 demonstrated a significant reduction in plaque area in Tg2576 AD mice. Together, these data suggest that SOBIN‐01 targets and stimulates the clearance of Aβ toxic oligomers.

